# Unveiling the Hidden Risk: Ticagrelor-Induced Bradyarrhythmias and Conduction Complications in ACS Patients—Case Series

**DOI:** 10.3390/jcdd13010007

**Published:** 2025-12-22

**Authors:** Aleksandra Gorzynska-Schulz, Damian Stencelewski, Ludmiła Daniłowicz-Szymanowicz, Monika Lica-Gorzynska, Agata Firkowska, Elżbieta Wabich

**Affiliations:** 1 Faculty of Medicine, Medical University of Gdansk, 80210 Gdansk, Poland; a.gorzynska@gumed.edu.pl (A.G.-S.); damian.sten777@gumed.edu.pl (D.S.); 2 Department of Cardiology and Electrotherapy, Medical University of Gdansk, 80210 Gdansk, Poland; ludwik@gumed.edu.pl; 3 Department of Cardiology, J.K. Lukowicz Memorial Specialist Hospital, 89600 Chojnice, Poland; monikalica@wp.pl; 4 II Department of Cardiology and Electrotherapy, University Clinical Center, 80210 Gdansk, Poland; agata.firkowska@gmail.com

**Keywords:** ticagrelor, bradyarrhythmias, atrioventricular block

## Abstract

Background: Ticagrelor is a reversible, direct inhibitor of the platelet adenosine diphosphate (P2Y12) receptor, widely used in combination with acetylsalicylic acid (ASA) as dual antiplatelet therapy (DAPT) in patients with acute coronary syndrome (ACS) to prevent cardiovascular events. Despite its well-established efficacy, ticagrelor may cause adverse effects ranging from common ones (e.g., bleeding, dyspnea) to rare but potentially serious reactions such as bradyarrhythmias. These rare events are likely related to elevated adenosine levels secondary to inhibition of the human equilibrative nucleoside transporter 1 (hENT1). Methods: We describe two clinical cases of ticagrelor-associated bradyarrhythmia observed in patients following ACS. Both cases were analyzed in terms of clinical presentation, ECG findings, management strategy, and outcomes after discontinuation of the drug. Results: The first case concerns a 67-year-old woman with non-ST-segment elevation myocardial infarction (NSTEMI) who developed complete atrioventricular block (third degree) with a 45 s asystolic pause and syncope. The second case involves a 67-year-old man with anterior ST-segment elevation myocardial infarction (STEMI) who experienced recurrent sinus pauses lasting up to 5 s. In both cases, symptoms resolved following ticagrelor discontinuation and theophylline administration. No recurrence of arrhythmia was observed after switching to prasugrel. Conclusions: Ticagrelor-induced bradyarrhythmias, although rare, represent an important and reversible adverse effect that clinicians should be aware of, particularly during the early post-ACS phase. Prompt recognition and drug withdrawal may prevent severe outcomes and avoid unnecessary interventions such as pacemaker implantation. Further studies are warranted to identify patient-specific risk factors predisposing to ticagrelor-related conduction disturbances.

## 1. Introduction

Ticagrelor is a reversible, direct-acting inhibitor of the adenosine diphosphate (ADP) receptor P2Y12 located on platelet membranes [1]. It inhibits platelet aggregation, suppresses inflammation, enhances adenosine activity, and provides cardioprotective effects [2]. Unlike some other antiplatelet agents, ticagrelor does not require metabolic activation, allowing it to achieve adequate blood concentrations within approximately two hours following oral administration, thereby making it a fast-acting and effective antiplatelet drug [3]. When used in combination with acetylsalicylic acid (ASA) as dual antiplatelet therapy (DAPT), ticagrelor significantly reduces the risk of cardiovascular events in adult patients with acute coronary syndrome (ACS), including unstable angina (UA), non-ST-elevation myocardial infarction (NSTEMI), and ST-elevation myocardial infarction (STEMI) [4]. Despite its proven efficacy, ticagrelor is associated with several adverse effects, the most notable and frequent being an increased risk of bleeding. Others include dyspnea, gout, renal impairment, and thrombotic thrombocytopenic purpura [5,6,7]. Bradyarrhythmias, including sinoatrial and atrioventricular conduction disturbances, are rare but potentially unpredictable complications of ticagrelor use. They are generally transient, often asymptomatic or mildly symptomatic, and are typically recorded during nighttime hours [6,8]. Although usually considered benign, such arrhythmias could become clinically significant, demanding discontinuation of ticagrelor and substitution with an alternative antiplatelet agent. Furthermore, bradyarrhythmias that arise during an ACS may also result from transient ischemia, making it challenging to determine whether ticagrelor is the direct cause, particularly during the early phase of therapy following an ACS event. In this report, we present two clinical cases involving ticagrelor-associated bradyarrhythmias. The first case describes a patient with NSTEMI who developed a complete atrioventricular block (AVB III) with a 45 s pause. The second case involves a patient with anterior-wall STEMI who experienced sinus pauses of up to 5 s after starting ticagrelor.

## 2. Materials and Methods

This is a single-center, observational case-series study describing two patients who experienced conduction disturbances and bradyarrhythmias following ticagrelor administration after myocardial infarction. We retrospectively identified two patients who were hospitalized in April 2024 at the Department of Cardiology in Chojnice, Poland, with a diagnosis of STEMI. Both patients underwent standard invasive treatment and received dual antiplatelet therapy including ticagrelor. During hospitalization, the occurrence of conduction abnormalities and bradyarrhythmias was documented in both cases.

Clinical data, including medical history, physical examination, laboratory findings, electrocardiographic (ECG) monitoring, and echocardiography results, were collected and analyzed. The temporal relationship between ticagrelor administration and the onset of rhythm disturbances was carefully assessed to evaluate a potential causal association.

## 3. Case Presentation

### 3.1. Case 1

**Figure 1:**
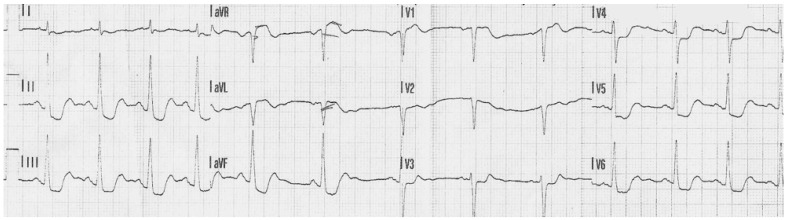
ST depression in most leads and significant ST elevation in the aVR lead.

A 67-year-old woman with a history of ovarian cancer, hyperlipidemia, nicotine dependence, and chronic obstructive pulmonary disease was admitted to the cardiology department due to chest pain, hypotension, and clinical signs of cardiogenic shock. Electrocardiography (ECG) showed ST-segment depression in most leads and marked ST elevation in aVR Figure 1. Based on these findings, a diagnosis of high-risk NSTEMI was made.

Laboratory tests revealed normal hemoglobin levels, an elevated white blood cell count, and slightly decreased electrolyte levels. However, low-density lipoprotein (LDL), troponin, and N-terminal pro-B-type natriuretic peptide (NT-proBNP) levels were significantly increased Table 1. Echocardiography showed a left ventricular ejection fraction (LVEF) of 40% with akinesia of the apical and mid-anterior, lateral, and septal segments, without left ventricular hypertrophy or significant valvular abnormalities.

Urgent coronary angiography followed by percutaneous coronary intervention (PCI) of the left main trunk with drug-eluting stent (DES) implantation led to rapid clinical improvement and resolution of cardiogenic shock. Before the procedure, the patient received a loading dose of 300 mg acetylsalicylic acid (ASA) and 180 mg ticagrelor, followed by maintenance doses of 75 mg ASA daily and 90 mg ticagrelor twice daily.

**Figure 2:**
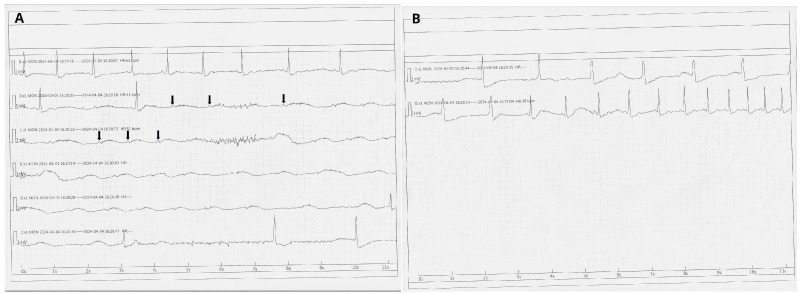
(**A**,**B**) Episode of complete atrioventricular block (AVB III) with a 45 s pause without an escape rhythm. Arrows indicate P waves.

On the fourth day of hospitalization, the patient developed a complete atrioventricular block (AVB III) with a 45 s pause with no escape rhythm (Figure 2A,B), accompanied by syncope and seizures. The episode resolved spontaneously once conduction resumed. Ticagrelor-induced bradyarrhythmia was suspected, as other causes of AV block, including hyperkalemia, acute kidney injury, or prior use of drugs affecting conduction (e.g., beta-blockers, calcium channel blockers, digoxin, amiodarone), were excluded. No evidence of ongoing ischemia, pulmonary congestion, or mechanical complications after myocardial infarction was found, indicating that PCI had successfully restored perfusion.

The suspected pathophysiology was likely adenosine-mediated, related to ticagrelor’s inhibition of the human equilibrative nucleoside transporter 1 (hENT1), which could increase adenosine concentration and may trigger transient bradyarrhythmias.

**Figure 3:**
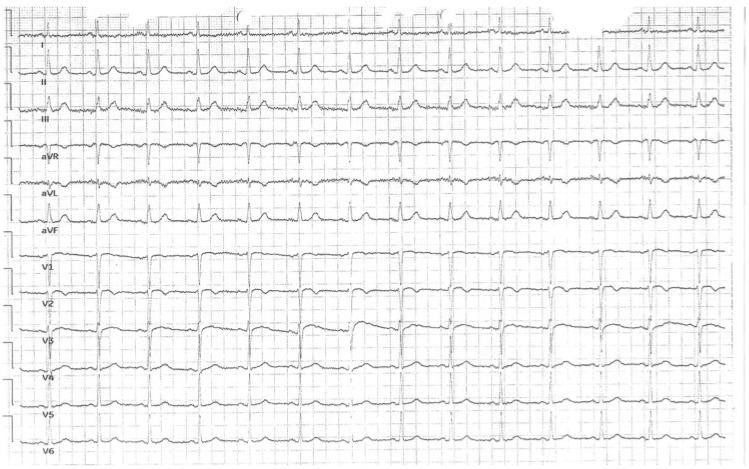
Inverted T waves in aVL and I leads with no other significant abnormalities.

Due to that fact, immediately after the pause, 200 mg of theophylline was administered intravenously to counteract the adenosine effect. Ticagrelor was discontinued, and therapy was switched to prasugrel, with no further occurrence of atrioventricular block. During the following days of hospitalization, no further episodes of bradycardia or conduction disturbances were observed. The final ECG showed only inverted T waves in leads I and aVL, without other abnormalities (Figure 3).

During the outpatient follow-up, the patient reported no symptoms of bradycardia, and 24 h Holter monitoring revealed no pauses or conduction blocks. The event was therefore attributed to ticagrelor use, therefore the patient was not qualified for permanent pacemaker implantation and continues to be closely monitored in the cardiology outpatient clinic.

### 3.2. Case 2

A 67-year-old man was admitted to the cardiology department with anterior STEMI diagnosis. Before hospitalization, he experienced typical chest pain for the first time in his life, lasting continuously for five hours without other accompanying symptoms. His medical history included COPD, tobacco use, alcohol dependence syndrome (in remission for three years), and episodes of epilepsy.

**Figure 4:**
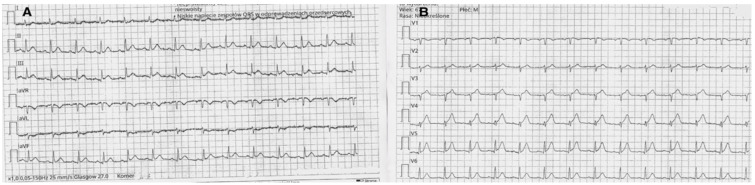
(**A**,**B**) Sinus rhythm 80 bpm with ST elevations in V3–V6 precordial leads, which suggest anterior-wall STE-ACS.

**Figure 5:**
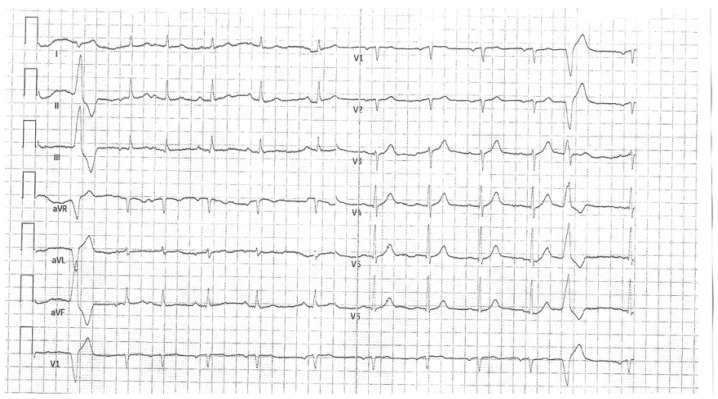
Post-PCI ECG with single premature ventricular beats and no pathological Q waves.

The ECG performed by the emergency medical team showed sinus rhythm at 80 bpm with ST-segment elevation in precordial leads V3–V6, confirming the diagnosis of anterior STEMI (Figure 4). The patient received 300 mg of ASA and 180 mg of ticagrelor before urgent coronary angiography, which revealed critical stenosis of the left anterior descending artery consistent with single-vessel disease. PCI with DES implantation was performed successfully, with no pathological Q waves on the post-PCI ECG [Figure 5].

Laboratory tests demonstrated significantly elevated low-density lipoprotein (LDL) and troponin levels (Table 2). Echocardiography revealed a left ventricular ejection fraction (LVEF) of 50%, subtle apical hypokinesis, mild left ventricular hypertrophy, and no significant valvular abnormalities. Post-procedure antiplatelet therapy included 75 mg ASA once daily and 90 mg ticagrelor twice daily.

**Figure 6:**
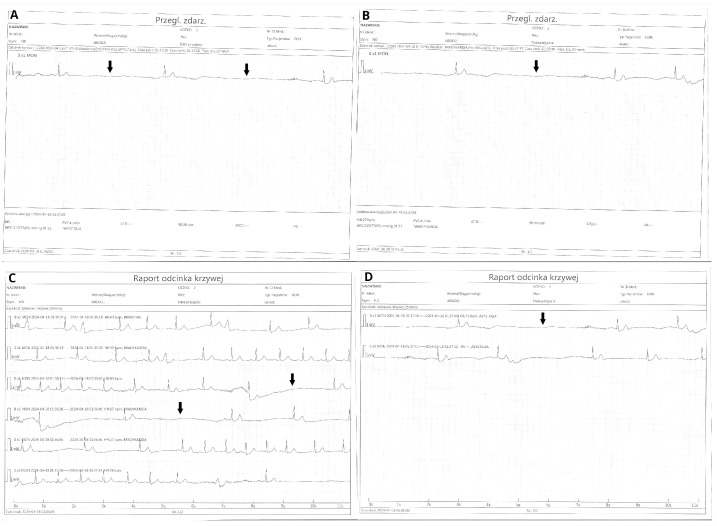
(**A**–**D**) Episodes of sinus arrest with pauses lasting approximately 3–5 s and a junctional escape rhythm. Arrows indicate pauses.

During the first night of hospitalization, the patient developed symptomatic sinus pauses lasting approximately 3–5 s, accompanied by junctional escape rhythm (Figure 6A–D). The patient was not receiving any drugs affecting conduction (e.g., beta-blockers, calcium channel blockers, digoxin, amiodarone), either prior to admission or during hospitalization. Moreover, given that coronary reperfusion was successful and no new ischemic changes were noted on the post-PCI ECG, the aforementioned pauses were suspected to be related to ticagrelor administration rather than a complication of myocardial infarction.

Therefore, 200 mg of theophylline was administered intravenously at a slow infusion rate, resulting in the restoration of a regular sinus rhythm. It should be emphasized that before the administration of theophylline, the sinus pauses recurred repeatedly; following its administration, no further pauses were observed. Hence, the decision to switch ticagrelor to prasugrel treatment was made.

Twelve weeks later, during follow-up coronary angiography and fractional flow reserve assessment, the patient reported no symptoms of bradycardia, and no pauses or atrioventricular block episodes were recorded.

## 4. Discussion

### 4.1. Ticagrelor—Principal Mechanism of Action

Ticagrelor is an oral antiplatelet drug that inhibits platelet aggregation by binding to the P2Y12 purinergic receptor on the platelet surface, thereby preventing the formation of a primary hemostatic clot [9,10]. Due to its therapeutic properties, ticagrelor is widely used in cardiology for the treatment of acute coronary syndromes (ACS) [1] as part of dual antiplatelet therapy (DAPT) [10] with acetylsalicylic acid (ASA) in patients undergoing percutaneous coronary intervention (PCI). Unlike clopidogrel and prasugrel, ticagrelor is not a prodrug, which results in a more rapid onset of antiplatelet activity compared with other P2Y12 inhibitors. This leads to a more predictable and consistent antiplatelet effect, unaffected by genetic variations in metabolic enzymes such as CYP2C19 [1,11,12]. It is also worth emphasizing that ticagrelor has an advantage over other P2Y12 antagonists, as it is the only one that reversibly inhibits the platelet P2Y12 receptor [1,10]. This reversible binding allows for a faster recovery of platelet function compared to irreversible inhibitors such as clopidogrel and prasugrel. This characteristic is particularly advantageous in patients requiring urgent surgical interventions, such as coronary artery bypass grafting (CABG), or when managing significant bleeding events. The PLATO trial demonstrated that ticagrelor, compared with clopidogrel, significantly reduces the risk of death from vascular causes, myocardial infarction, or stroke, without a substantial increase in bleeding risk [12,13,14]. Therefore, ticagrelor is the preferred P2Y12 inhibitor in the management of ACS, particularly STEMI, according to current clinical guidelines [1].

### 4.2. Adverse Reactions to Ticagrelor

**Figure 7:**
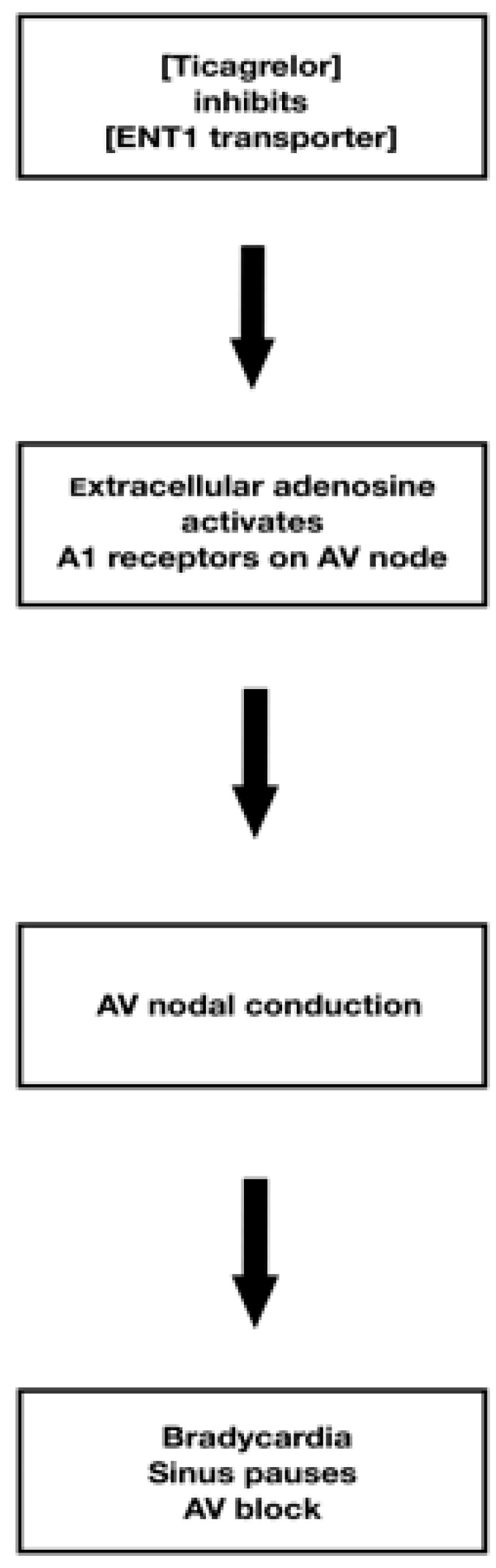
Arrythmogenic mechanism of action of Ticagrelor administration.

The most common adverse effect of ticagrelor is an increased tendency to bleed, particularly from the gastrointestinal tract, gums, and upper respiratory tract, occurring in approximately 11–12% of patients [15]. Other common adverse reactions (≥1% to <10%) include dyspnea (up to 13.8%), nausea (approximately 2.5%), renal impairment (up to 3%), gout (2–4%), vomiting (2–3%), and dizziness (around 3%) [9,13,16]. Bradyarrhythmias, such as atrioventricular block (AVB) and sinus pauses, are rare (<1%) but potentially serious complications associated with ticagrelor, as initially reported in the PLATO trial [17]. However, other studies have documented a higher incidence, with rates reaching up to 5.8%, highlighting variability in the reported frequency of these events [17,18]. Although the exact mechanism by which ticagrelor affects cardiac conduction and sinoatrial (SA) nodal automaticity remains unclear, several plausible pathways have been proposed [1,11,14]. A key mechanism involves ticagrelor-induced inhibition of the equilibrative nucleoside transporter 1 (ENT1), resulting in increased plasma levels of adenosine [1,3,6,8]. By blocking ENT1, which facilitates the cellular uptake of adenosine, ticagrelor prolongs the extracellular presence of this purine nucleoside [1,2,15]. Normally, adenosine—produced during ATP or ADP metabolism—is rapidly transported into cells via ENT1 and metabolized by cytoplasmic enzymes such as adenosine deaminase and adenosine kinase [1,15]. Because adenosine has an extremely short half-life of only a few seconds, its concentration depends on a very rapid balance between production and clearance. Ticagrelor reduces adenosine clearance by inhibiting ENT1-mediated cellular uptake, and in such a fast-turnover system, even minor changes in clearance lead to an immediate rise in extracellular adenosine. This, in turn, potentiates A1-receptor-mediated slowing of sinoatrial and atrioventricular nodal conduction, which explains the clinically observed bradycardia and episodes of heart block reported in several studies [2,7,9,11,14]. Furthermore, by blocking the P2Y12 receptor, ticagrelor may further elevate local adenosine concentrations [1,3,6,8,10,13,19]. Elevated adenosine, through the activation of A1 receptors in the SA node, slows the heart rate and may result in bradycardia or even sinus pauses. Adenosine exerts its cardiovascular effects primarily through A1 receptors located on cardiomyocytes within the supraventricular conduction system—including the SA node (Figure 7), atrial tissue, and atrioventricular (AV) node—while having minimal effects on the ventricular myocardium [7,13,14]. Upon binding to A1 receptors, adenosine inhibits adenylate cyclase, leading to reduced cyclic adenosine monophosphate (cAMP) levels. This cascade produces a negative chronotropic effect by slowing the depolarization rate of pacemaker cells and a negative dromotropic effect by reducing conduction through the AV node. In susceptible individuals, these effects may be pronounced enough to cause high-grade AV block, including complete (third-degree) AVB [9,10,15]. Notably, these same properties underlie the clinical use of adenosine in terminating reentrant supraventricular tachyarrhythmias (SVTs) [1]. Additionally, ticagrelor’s direct modulation of the SA node may reduce automaticity, further contributing to bradyarrhythmias [8,14]. Other potential etiologies of bradyarrhythmias in patients following ACS are summarized in Table 3 [17,18].

### 4.3. Management of Ticagrelor-Induced Pauses

Ticagrelor has been associated with asymptomatic sinus pauses in patients receiving dual antiplatelet therapy [12,13,14]. As demonstrated in our cases, such pauses may become hemodynamically significant and potentially life-threatening. To the best of our knowledge, these are the first reported instances in the literature of long-lasting pauses (up to 45 s) following ticagrelor administration, likely mediated by adenosine’s effect on the sinoatrial node. According to the literature, the highest risk of bradyarrhythmic episodes [7,20] after ticagrelor administration occurs within the first week following an ACS. Therefore, close monitoring is essential, particularly during the initial days after an ACS. Proactive care is important because, in the event of bradyarrhythmic episodes, timely intervention can be implemented [7,15,21]. In cases of bradyarrhythmic disturbances, administration of 200 mg of theophylline intravenously has been proposed [10]. Theophylline, a methylxanthine derivative, is not a direct antidote to ticagrelor but reverses bradycardic effects mediated by elevated adenosine levels [1,2,9,13,14]. It acts as a reversing agent specifically in the context of ticagrelor administration; therefore, theophylline would be ineffective with other P2Y12 antagonists [21]. Theophylline functions as a non-selective phosphodiesterase (PDE) inhibitor, leading to increased intracellular cAMP levels. This results in the inhibition of adenosine receptors, particularly A1 receptors, which mediate adenosine’s effects on the heart. By blocking these receptors, theophylline counteracts adenosine’s actions, thereby increasing the heart rate and improving conduction through the atrioventricular node. This mechanism makes theophylline effective in situations where bradycardia or conduction abnormalities occur due to excessive adenosine activity, such as that induced by ticagrelor [6,8,10,21]. Management in the presented cases focused on reversing ticagrelor’s effects on the cardiac conduction system. Notably, switching from ticagrelor to an alternative P2Y12 antagonist resulted in clinical improvement and resolution of bradyarrhythmias. Since bradyarrhythmic effects have not been reported during treatment with prasugrel or clopidogrel, these agents can serve as alternatives to ticagrelor [2,11,15,21]. Follow-up confirmed that arrhythmias did not recur, strongly suggesting a causal relationship between ticagrelor and the observed bradyarrhythmias. Prior to hospitalization, the patients were not taking medications that could affect cardiac conduction system automaticity. Moreover, after ACS, no new drugs known to affect cardiomyocyte activity were introduced [7,21,22]. In clinical practice, pharmacological agents other than ticagrelor can thus be ruled out as potential causes of sinus pauses or third-degree AV block [21]. Additionally, the administration of theophylline and rapid cessation of arrhythmic events provide compelling evidence supporting the probable role of ticagrelor-induced bradyarrhythmia [7]. These cases are clinically important because unawareness of bradycardia as a potential side effect of ticagrelor may lead to unnecessary pacemaker implantation, despite bradycardia being reversible. This study emphasizes ticagrelor’s potential bradyarrhythmic effects and highlights a crucial consideration in the management of patients receiving this drug.

## 5. Conclusions

The presented clinical cases highlight a rare but potentially life-threatening adverse effect of ticagrelor administration. Ticagrelor-induced pauses are generally considered short and asymptomatic; however, they can be prolonged and clinically significant, as demonstrated in these patients. Clinicians should be aware of this potential side effect to prevent severe complications and avoid unnecessary pacemaker implantation.

## Figures and Tables

**Table 1:** Laboratory tests.

Laboratory Parameter	Reference Range	Hospitalization
Red Blood Cells (T/L)	3.85–5.2	4.56
Hemoglobin (g/dL)	11.8–15.8	14.2
Platelets (G/L)	160.0–370.0	279.0
White Blood Cells (G/L)	3.6–10.5	16.86
Low-Density Lipoprotein (mg/dL)	<55	280
Sodium (mmol/L)	136–145	128
Potassium (mmol/L)	3.5–5.1	3.1
Ultrasensitive troponin I (µg/L)	<0.0156	0.1770
Creatinine (mg/dL)	0.50–1.20	0.97
Thyroid-Stimulating Hormone (mlU/L)	0.35–4.94	2.24
N-terminal pro B-type Natriuretic Peptide (pg/mL)	<125.00	733.20

**Table 2:** Laboratory tests.

Laboratory Parameter	Reference Range	Hospitalization
Red Blood Cells (T/L)	4.0–5.65	5.13
Hemoglobin (g/dL)	12.5–17.2	15.6
Platelets (G/L)	160.0–370.0	211.0
White Blood Cells (G/L)	3.6–10.5	10.0
Low-Density Lipoprotein (mg/dL)	<55	145
Sodium (mmol/L)	136–145	137
Potassium (mmol/L)	3.5–5.1	4.1
Ultrasensitive troponin I (µg/L)	<0.0342	13.8980
Creatinine (mg/dL)	0.70–1.30	0.70
Thyroid-Stimulating Hormone (mg/dL)	0.35–4.94	1.05

**Table 3:** Causes of bradyarrythmias in Post-STEMI patients.

Cause	Estimated Prevalence (%)
Ischemia of infarction of conduction system (e.g., AV node, His bundle)	30–40%
Increased vagal tone (especially in inferior MI)	15–25%
Electrolyte imbalances (e.g., hyperkalemia)	5–10%
Use of AV nodal blocking drugs (e.g., beta-blockers, calcium channel blockers, digoxin)	10–20%
Reperfusion injury (e.g., transient AV block after PCI)	5–10%
Age-related degeneration (fibrosis of conduction system)	5–10%
Ticagrelor-induced sinus pauses	<6%
Hypothyroidism	<2%

## Data Availability

Data are available upon request.
